# Validity and reliability testing of the Thai version of the emPHasis-10 questionnaire for patients with pulmonary arterial hypertension

**DOI:** 10.1186/s41687-026-01017-0

**Published:** 2026-02-20

**Authors:** Sahachat Aueyingsak, Kanokporn Khankaew, Wimolporn Teerawattananant, Parinyaporn Singsangtam, Aroonsri Sanmueang, Montri Yasud, Nichamon Ekphaphan, Burabha Pussadhamma

**Affiliations:** 1 Department of Physical Therapy, College of Health Sciences, Christian University of Thailand, Nakhon Pathom, Nakhon Pathom Province Thailand; 2 Queen Sirikit Heart Center of the Northeast, Faculty of Medicine, Khon Kaen University, Khon Kaen Province, Thailand; 3 Department of Medicine, Faculty of Medicine, Khon Kaen University, Khon, Kaen Province Thailand

**Keywords:** emPHasis-10 questionnaire, Thai, Quality of life, Pulmonary arterial hypertension, Pulmonary hypertension

## Abstract

**Background:**

The emPHasis-10 questionnaire is a specific tool for assessing health-related quality of life (HRQoL) in pulmonary arterial hypertension (PAH). We conducted the cross-cultural validation of the Thai emPHasis-10 questionnaire with an assessment of its validity and reliability in patients with PAH.

**Methods:**

The original English emPHasis-10 questionnaire was translated into Thai using a forward and backward translation process. Content validity was assessed by five experts using the Item-Objective Congruence (IOC) index, and reliability was assessed by 20 patients with PAH using Cronbach’s alpha for internal consistency and the intraclass correlation coefficient (ICC) for test-retest reliability. All patients had a confirmed diagnosis of PAH by right heart catheterization, and baseline clinical and hemodynamic characteristics were collected.

**Results:**

For Thai emPHasis-10 questionnaire, the IOC value was 0.94 (range 0.80 to 1.00). Among all patients, 19 (95%) were female, and the mean age±standard deviation (SD) was 46.8 ± 14.2 years, and 17 (85%) were at World Health Organization functional class II. Idiopathic PAH, PAH associated with connective tissue disease, and PAH associated with congenital heart disease were classified in 7, 6, and 7 patients, respectively. The mean baseline mean pulmonary arterial pressure and pulmonary vascular resistance were 55.1 ± 19.5 mmHg and 13.1 ± 8.4 Wood units, respectively. The mean ± SD of the Thai emPHasis-10 score was 18 ± 10. Cronbach’s alpha and ICC values were 0.925 and 0.996 (95% confidence interval 0.991 to 0.999), respectively.

**Conclusion:**

The Thai emPHasis-10 questionnaire has acceptable validity and reliability. This tool can be used as an additional measure for PAH severity assessment.

**Supplementary Information:**

The online version contains supplementary material available at 10.1186/s41687-026-01017-0.

## Introduction

Pulmonary arterial hypertension (PAH) is a specific subgroup of pulmonary hypertension (PH), being an uncommon disorder, and has been found to lead to limited survival of patients around the globe [1, 2]. Almost all PAH patients experience dyspnea and fatigue, which can lead to mental problems and a lower health-related quality of life (HRQoL) [3]. Moreover, these symptoms make it difficult for physicians to examine and diagnose the condition, posing a significant problem for prognostication. One study in patients with PH from the United Kingdom (UK) found that PH has a huge impact on their overall quality of life (QoL), and improvement of QoL is the priority of patients’ hopes to gain from treatment [4]. Currently, the use of HRQoL questionnaires or patient-reported outcome measures (PROMs) is being encouraged as a necessary tool in assisting with the evaluation of PAH patients [5].

Several PROMs are available to assess HRQoL in pulmonary hypertension, including generic tools such as the SF‑36 and PAH‑specific instruments such as CAMPHOR and emPHasis‑10. The emPHasis‑10 was chosen for this study because it is a brief, 10‑item, PAH‑specific questionnaire that is easy for patients to complete and practical for routine clinical use. Previous studies have shown that the much shorter emPHasis‑10 correlates strongly with the more complex and time‑consuming CAMPHOR questionnaire and provides comparable longitudinal information on HRQoL and clinical status in patients with PAH and chronic thromboembolic pulmonary hypertension, while being easier to implement in daily practice [6]. Based on these advantages and the absence of a Thai version, emPHasis‑10 was considered the most suitable instrument for cross‑cultural adaptation in Thai patients with PAH.

The emPHasis-10 questionnaire had a positive correlation with the World Health Organization (WHO) functional class [7]. The questionnaire has been utilized in PAH risk stratification and has shown significant correlations with exercise tolerance, cardiac biomarkers, and hemodynamics among many subgroups of patients with PAH [8–11]. Furthermore, it has also been found to be an independent predictor of mortality and is able to further differentiate survival among PAH patients with WHO functional class III [10]. Although the emPHasis-10 questionnaire has provided significant additional value for PAH patient assessment, one limitation is that it was initially developed in English and for self-evaluation, which may pose challenges when used in different languages and cultural contexts. Therefore, translation, cross-cultural adaptation, and assessment of the psychometric properties of the emPHasis-10 questionnaire in languages other than English are crucial for its applicability in diverse cultural contexts, and many countries have undertaken the translation of the emPHasis-10 questionnaire to facilitate its use in their own populations [12–16].

In Thailand, there is currently no established and validated HRQoL questionnaire specifically designed for patients with PAH. We hence aimed to translate and validate a Thai-specific questionnaire for PAH patients. Therefore, this study was conducted to determine the content validity and rater reliability of the newly translated and adapted Thai version of the emPHasis-10 questionnaire.

## Method

**Fig. 1:**
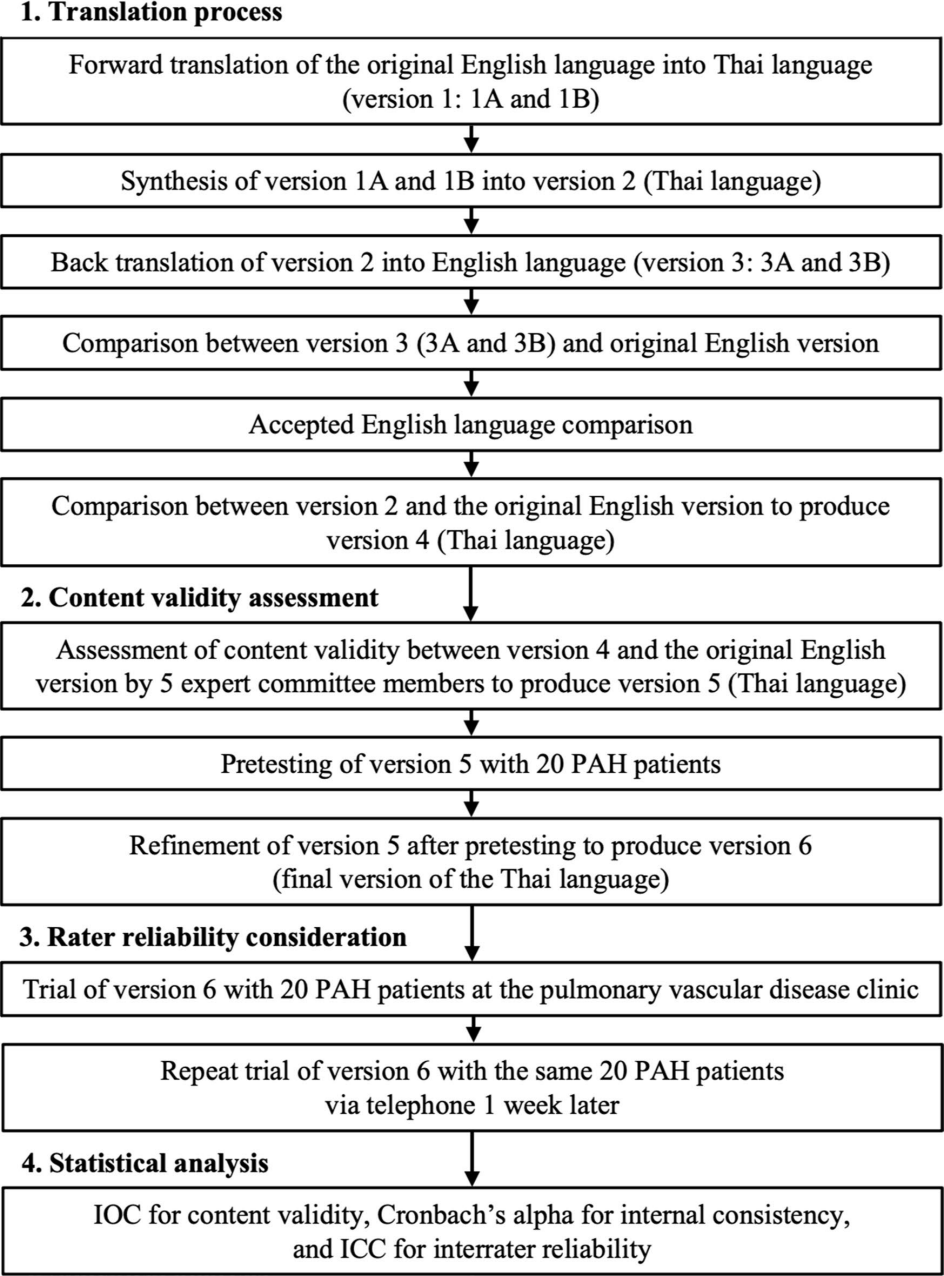
Translation process from the original english emPHasis-10 questionnaire to Thai. ICC denotes intraclass correlation coefficient; IOC, item-objective congruence; PAH, pulmonary arterial hypertension

There are four steps in the procedure, as depicted in Fig. 1. All steps were carried out following the approval of the study by the authors’ affiliated Center for Ethics in Human Research at Khon Kaen University, in accordance with the principles of the Declaration of Helsinki (HE651076). Permission to translate and validate the Thai questionnaire was granted by the Pulmonary Hypertension Association UK. The questionnaire comprises 10 items, and patients can respond using a scale of 0 to 5 to describe their HRQoL in relation to their symptoms. A consecutive convenience sample of 20 eligible patients with PAH who attended the pulmonary vascular disease clinic and provided informed consent was included in the study.

The design and validation of PRO measures, including cross‑cultural adaptations, are guided by international recommendations such as the COSMIN guidelines [17] and the FDA PRO guidance [18], which emphasize rigorous evaluation of content validity and test-retest reliability.

### Translation process

#### Forward translation

Forward translation from English to Thai was carried out by two independent bilingual translators who primarily use Thai as their main language. One of the translators was a cardiologist (clinician; BP), while the other was an English-language expert from outside the medical field (linguist). The two translators independently translated the original emPHasis-10 version into Thai, resulting in two separate versions (1 A and 1B).

#### Synthesis

The two translators (BP and SA) synthesized Thai versions 1 A and 1B by comparing each of their translations and reaching a consensus on the phrasing to synthesize the second Thai version of the questionnaire (version 2).

#### Back-translation

The back-translation (Thai to English) was conducted by two native English speakers who can read Thai and had never seen the original emPHasis-10 questionnaire. They performed a back-translation of version 2 into English. This step resulted in two back-translated versions (3 A and 3B).

#### Comparison of the original version and the two back-translated versions

Assessors (BP and SA) compared versions 3 A and 3B with the original English version for conformity and meaning. If the back-translated versions matched closely with the original English version, then the second Thai version (version 2) was deemed appropriate for the next step.

#### Comparison between version 2 and the original english version to produce version 4 (Thai language)

The translation process team reviewed the accuracy and comprehension of the second Thai version (version 2) by comparing it with the original English version. This step determined the validity of the second Thai version (version 2) to further produce the third Thai version (version 4).

### Content validity assessment

#### Expert committee review

The original English version and the third Thai version (version 4) were reviewed for repetitive equivalent meanings and content validity using the Item-Objective Congruence (IOC) index. This review was conducted by a committee of five experts, including a cardiologist, a pulmonologist, a rheumatologist, a respiratory physical therapist, and a cardiac physical therapist. The IOC index, applied by these specialists, involved an evaluation of each question on an ordinal scale (+ 1, 0, or -1) to indicate its correlation with the objectives of the questions. BP and SA collected feedback from the committee’s assessments, and if necessary, the questionnaire was revised and updated accordingly. This step resulted in the fourth Thai version (version 5).

#### Pretesting and refinemnet

The fourth Thai version (version 5) was trialed in 20 patients with PAH to determine whether the items in the emPHasis-10 questionnaire were clear and easy to use in a clinical setting. During this phase, no modifications to the questionnaire were necessary. However, the authors added descriptors to enhance its clarity, resulting in the final version of the Thai emPHasis-10 questionnaire (version 6).

### Rater reliability consideration

The emPHasis-10 questionnaire was designed for self-assessment by patients with PAH. Its reliability was assessed in terms of both internal consistency and interrater agreement among patients with PAH. This assessment was based on Cronbach’s alpha for internal consistency and the intraclass correlation coefficient (ICC) for test-retest reliability, following a study conducted by Mustafaoglu and colleagues [19]. The study included a sample of 20 participants who were invited after being diagnosed with PAH by right heart catheterization. To assess the test-retest reliability, all participants were questioned using the final version of the Thai emPHasis-10 questionnaire twice. The first assessment was conducted at the pulmonary vascular disease clinic of Srinagarind Hospital and Queen Sirikit Heart Center of the Northeast, Khon Kaen University, after signing informed consent, during which a cardiologist and PAH specialist (BP) conducted interviews with each individual participant. Demographic and baseline characteristics, including age, sex, body mass index, education level, type of PAH, WHO functional class, six-minute walk distance (6MWD), N-terminal pro-brain natriuretic peptide (NT-proBNP) levels, and right heart catheterization data, were also collected during the first assessment. The second assessment of the final version of the Thai emPHasis-10 questionnaire was conducted by SA via telephone one week later, aiming to minimize recall bias.

### Statistical analysis

In line with Anthoine et al. [20], there is currently no internationally accepted consensus on minimum sample size requirements for validation studies of patient‑reported outcomes, and most studies rely on pragmatic sample sizes or subject‑to‑item rules. In our study, PAH is a rare disease with limited numbers of eligible patients. Therefore, we enrolled all eligible and consenting patients during the study period (*n* = 20), which is consistent with common practice in rare‑disease validation studies. Consequently, a formal a priori sample size calculation was not performed. Descriptive analysis of the data was conducted using percentages, means, standard deviations (SD), minimum, and maximum values to describe the demographic characteristics of the patients. Content validity was evaluated using the IOC index. Internal consistency and test-retest reliability of the Thai emPHasis-10 questionnaire were measured using Cronbach’s alpha and the ICC model for the total score, respectively. The validity and reliability of the questionnaire were assessed using the IBM SPSS statistical package (version 28.0).

## Results

### Participants

**Fig. 2:**
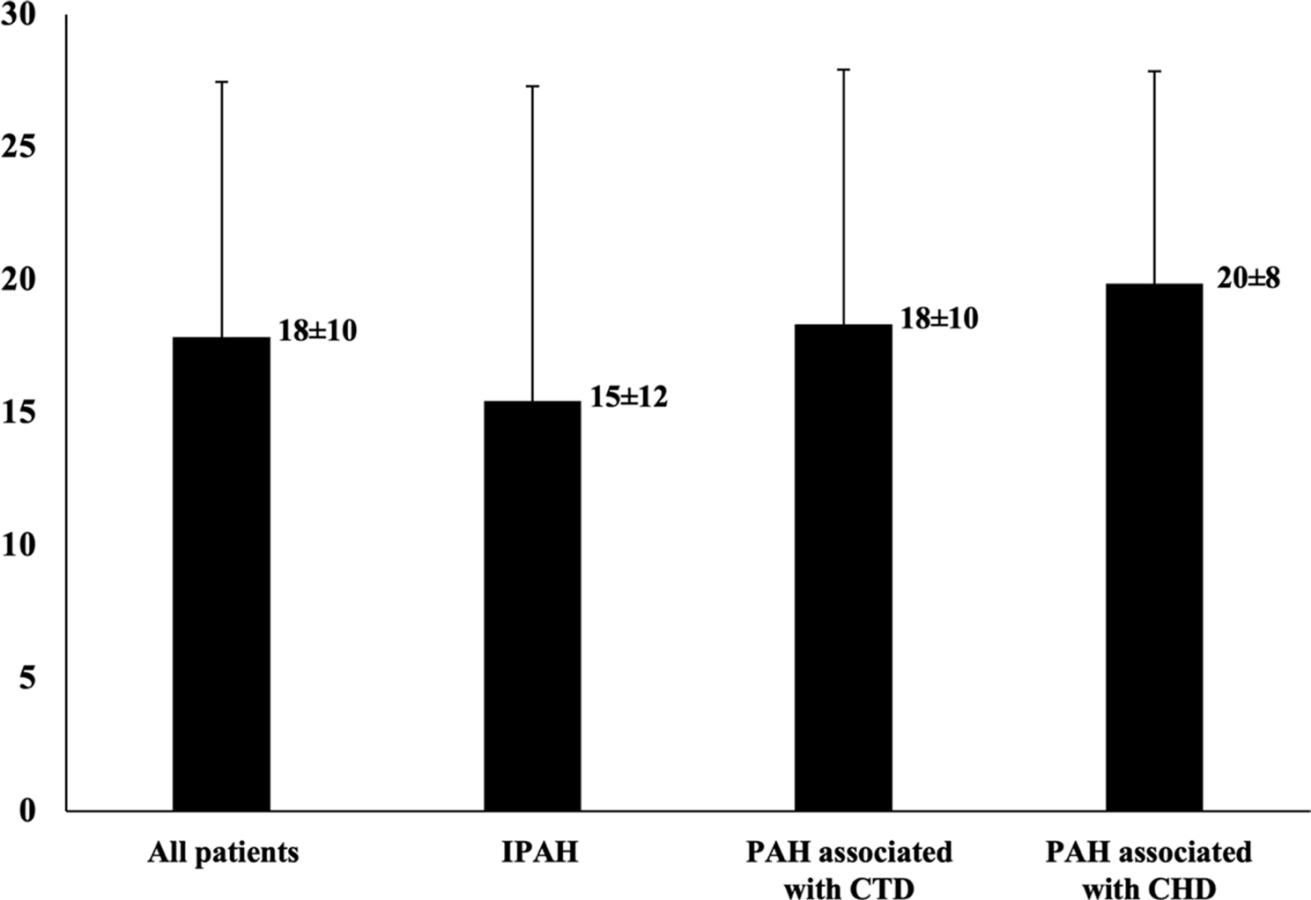
Thai emPHasis-10 score among participants. Data were presented as mean±standard deviation. CHD denotes congenital heart disease; CTD, connective tissue disease; IPAH, idiopathic pulmonary arterial hypertension; PAH, pulmonary arterial hypertension

Characteristics of 20 participants are presented in Table 1. Among them, the mean age ± SD was 46.8 ± 14.2 years, and the majority were female (19, 95%). Most of the participants had graduated below university level (14, 70%) and were covered by the Universal Coverage (UC) medical scheme of the country (12, 60%). Idiopathic PAH and PAH associated with congenital heart disease (CHD) each equally comprised 7 participants (35%), while PAH associated with connective tissue disease (CTD) comprised 6 participants (30%). Most of the participants were mildly symptomatic with WHO functional class II during the baseline assessment (17, 85%), whereas 2 participants (10%) were at WHO functional class III. Right heart catheterization showed typical features of PAH, with the mean ± SD of mean pulmonary arterial pressure (mPAP), pulmonary arterial wedge pressure (PAWP), and pulmonary vascular resistance (PVR) being 55.1 ± 19.5 mmHg, 7.8 ± 3.0 mmHg, and 13.1 ± 8.4 Wood units, respectively. The baseline mean ± SD of the Thai emPHasis-10 score among all participants was 18 ± 10, and the baseline mean ± SD of the Thai emPHasis-10 score in the subgroups idiopathic PAH, PAH associated with CTD, and PAH associated with CHD were 15 ± 12, 18 ± 10, and 20 ± 8, respectively (*p* = 0.705) (Fig. 2).

**Table 1:** Baseline characteristics of participants

Characteristics	*N* = 20
Demographic characteristics	
Age (y)	46.8 ± 14.2
Female	19 (95)
Weight (kg)	46.9 ± 8.0
Height (cm)	153.7 ± 7.4
BMI (kg/m^2^)	19.8 ± 2.6
Education	
Primary school	3 (15)
Junior high school	7 (35)
Senior high school	4 (20)
University	6 (30)
Health care coverage	
UC	12 (60)
CSMBS	5 (25)
SSS	3 (15)
PAH characteristics	
Type of PAH	
Idiopathic PAH	7 (35)
PAH associated with CTD	6 (30)
PAH associated with CHD	7 (35)
WHO functional class	
I	1 (5)
II	17 (85)
III	2 (10)
6MWD (m)	340 ± 78
NT-proBNP (pg/mL)	1,285 ± 1,478 842.5 (284.0-1684.5)
Right heart catheterization	
mRAP (mmHg)	6.7 ± 3.1
mPAP (mmHg)	55.1 ± 19.5
PAWP (mmHg)	7.8 ± 3.0
Cardiac output (L/min)	3.5 ± 1.2
Cardiac index (L/min/m²)	2.6 ± 0.8
PVR (Wood units)	13.1 ± 8.4

Data were presented as mean±standard deviation or median (interquartile range) or N (%) as appropriated

6MWD denotes six-minute walk distance; BMI, body mass index; CHD, congenital heart disease; CSMBS, civil servant medical benefit scheme; CTD, connective tissue disease; mPAP, mean pulmonary arterial pressure; mRAP, mean right atrial pressure; NT-proBNP, N-terminal pro-brain natriuretic peptide; PAH, pulmonary arterial hypertension; PAWP, pulmonary arterial wedge pressure; PVR, pulmonary vascular resistance; SSS, social security scheme; UC, universal coverage; WHO, World Health Organization

### Translation process

A final version of the Thai emPHasis-10 questionnaire (in the supplement) was finalized following the guidelines of cross-cultural adaptation [21], with descriptors for every item were later translated and validated to enhance user clarity.

### Content validity assessment

For all 10 items, the content validity of the Thai emPHasis-10 questionnaire achieved an IOC value of 0.94 (range 0.80 to 1.00). This value indicates excellent validity (Table 2).

**Table 2:** The content validity (expert committee review) and internal consistency (Cronbach’s alpha) of each item of Thai emPHasis-10 questionnaire

Item	Description		IOC index	Cronbach’s alpha
1	I am not frustrated by my breathlessness	I am very frustrated by my breathlessness	1	0.91
2	Being breathless never interrupts my conversations	Being breathless always interrupts my conversations	1	0.91
3	I do not need to rest during the day	I always need to rest during the day	0.8	0.91
4	I do not feel exhausted	I always feel exhausted	0.8	0.92
5	I have lots of energy	I have no energy at all	1	0.92
6	When I walk up one flight of stairs I am not breathless	When I walk up one flight of stairs I am very breathless	1	0.92
7	I am confident out in public places/crowds despite my PH	I am not confident at all in public places/crowds because of my PH	1	0.91
8	PH does not control my life	PH completely controls my life	1	0.90
9	I am independent	I am completely dependent	0.8	0.91
10	I never feel like a burden	I always feel like a burden	1	0.92

IOC denotes item-objective congruence; PH, pulmonary hypertension

### Rater reliability consideration

Both internal consistency and test-retest reliability among patients with PAH, as assessed using the Thai emPHasis-10 questionnaire to evaluate the patients’ HRQoL, showed excellent agreement, which the internal consistency had a Cronbach’s alpha value of 0.925, and the test-retest reliability had an ICC value of 0.996 (95% confidence interval (CI): 0.991 to 0.999) (Table 2).

## Discussion

We are the first group in Thailand to conduct an official translation of the emPHasis-10 questionnaire from English to the Thai language. In our single-center study, five experts from different fields were involved in the content validity assessment, and twenty patients with PAH were participated in the rater reliability assessment. In this pilot sample, the Thai emPHasis‑10 showed good content validity (IOC = 0.94) and high internal consistency and test–retest reliability (Cronbach’s alpha = 0.925; ICC = 0.996 (95% CI 0.991 to 0.999)). However, these estimates were obtained from a small, relatively homogeneous sample and should be interpreted with caution.

The emPHasis-10 questionnaire was initially developed in English, which can pose a challenge for patients who cannot read English. In its original survey in English conducted in patients with various etiologies of PH, a Cronbach’s alpha value of 0.90 and an ICC value of 0.95 were achieved [7]. In recent times, numerous health professionals have undertaken efforts to translate it into various languages to accommodate individual patients. This process, known as linguistic validation, is designed to ensure that the translated questionnaire maintains high quality and yields consistent results, in line with the original master questionnaire in English. In a study conducted in Turkey involving various groups of patients with PH, a high Cronbach’s alpha value of 0.98 and an ICC value of 0.97 were reported [13]. A study conducted in Italy among patients with PH showed that the Italian version of the emPHasis-10 questionnaire was reliable with a Cronbach’s alpha value of > 0.9 [14]. A recent study of Japanese patients with various PAH and chronic thromboembolic pulmonary hypertension (CTEPH) showed a slightly lower degree of validity and reliability, with a Cronbach’s alpha value of 0.86 along with an ICC value of 0.89 [15]. A recent study of Chinese patients with PAH associated with CTD showed a high Cronbach’s alpha value of 0.95 [16]. Compared with prior studies, the reliability coefficients observed for the Thai emPHasis‑10 are of similar magnitude to those reported in Turkish, Italian, Japanese, and Chinese versions [13–16]. However, previous validations generally included larger samples (ranging from about 70 to over 200 patients) and, in some cases, incorporated additional psychometric analyses such as factor analysis, whereas our study evaluated only content validity and test–retest reliability in a small single‑center pilot sample of 20 patients. These methodological and sample‑size differences mean that our findings should be considered preliminary, and direct comparisons with other language versions must be interpreted with caution.

Although previous validations of the emPHasis-10 questionnaire were conducted in various etiologies of PH and diverse cultural backgrounds, the results of rater reliability, including our findings, were excellent, except for a study from Japan which showed a good level [15]. This harmony could indicate the broad utility of the emPHasis-10 questionnaire for patients with any group of PH, and could also reflect the similar impact of cardiorespiratory disturbance from PH on patients’ perspectives irrespective of the specific etiology of PH.

It is possible that comorbidities or associated conditions in individual patients with PAH could have an impact on patients’ perspectives on their own illness with PAH and lead to varying responses on the HRQoL questionnaire, although the severity of their PAH is similar. In our study, there was no significant difference in the Thai emPHasis-10 questionnaire scores among the three subgroups of patients. However, patients with idiopathic PAH, who had fewer comorbidities, reasonably reported a numerically lower score compared with the other two subgroups of patients with PAH who had comorbid conditions. To our knowledge, no previous studies have examined this issue. The small sample size in our study makes it insufficient to draw a firm conclusion, and we recommend a larger study to address this issue in the future.

### Limitations

Our study contained several limitations. (1) The number of patients was relatively small (*n* = 20) compared with prior validation studies, which restricted our statistical analysis to reliability and content validity. (2) The sample was relatively homogeneous: most participants were female (95%) and had only mild to moderate PAH severity (WHO functional class II); therefore, the generalizability of our findings to male patients and to those with more severe PAH is limited. (3) All enrolled patients had PAH; thus, the use of the Thai emPHasis-10 questionnaire in patients with non-PAH conditions might yield different results. However, as prior studies were conducted in various groups of patients with PH and still achieved excellent reliability, and since cardiorespiratory consequences among each PH etiology are quite similar, we assume that this questionnaire could practically be used in other group of PH patients. (4) A correlation study between the Thai emPHasis-10 and other key parameters (6MWD, NT-proBNP) was not conducted in this pilot study. However, these correlations have been well established in previous studies [8–11]. (5) Other general instruments for HRQoL assessment, such as the EuroQol five-dimensions (EQ-5D) questionnaire, were not assessed in our study. Therefore, a correlation between the Thai emPHasis-10 questionnaire and other widely used HRQoL questionnaires could not be demonstrated.

To address these limitations, future studies with a larger sample size (planned *n* ≈ 100) will be conducted. These studies will employ advanced psychometric methods, including exploratory factor analysis and Rasch modeling, to fully assess construct validity. Furthermore, we will assess convergent validity by examining correlations with key clinical anchors, specifically 6MWD, NT‑proBNP, and WHO functional class, to confirm the instrument’s clinical utility and psychometric robustness in the Thai population.

## Conclusion

The Thai emPHasis-10 questionnaire demonstrated acceptable validity and reliability in this pilot study, but further validation in larger, diverse samples is required. This tool is user-friendly and provides an effective approach to measure the perspectives of patients suffering from PAH. Use of the Thai emPHasis-10 questionnaire may serve as an additional parameter to assess PAH severity.

## Supplementary Information

Below is the link to the electronic supplementary material.

Supplementary Material 1

## Data Availability

Data will be made available on reasonable request.
