# Supplementary material for: Validity and reliability testing of the Thai version of the emPHasis-10 questionnaire for patients with pulmonary arterial hypertension
Source: J Patient Rep Outcomes. 2026 Feb 20;10:30. doi: 10.1186/s41687-026-01017-0 (PMC12923680; doi:10.1186/s41687-026-01017-0)
Supplement: Supplementary file 1 — Supplementary Material 1 [file 41687_2026_1017_MOESM1_ESM.pdf]

แบบสอบถามเอ็มฟะซิส 10 (emPHasis-10 questionnaire)

แบบสอบถามนี้ออกแบบมาเพื่อประเมินผลกระทบของภาวะความดันหลอดเลือดปอดสูงต่อชีวิตของท่าน กรุณาตอบแบบสอบถามโดยทำเครื่องหมายถูก (✓) บนช่องตัวเลข "เพียงช่องเดียว" ซึ่งตรงกับความรู้สึกของท่านในระยะนี้มากที่สุด

ข้าพเจ้าไม่รู้สึกหงุดหงิดจากอาการหายใจไม่อิ่ม	0 1 2 3 4 5	ข้าพเจ้ารู้สึกหงุดหงิดจากอาการหายใจไม่อิ่มเป็นอย่างมาก
อาการหายใจไม่อิ่มไม่เคยขัดจังหวะการสนทนาของข้าพเจ้า	0 1 2 3 4 5	อาการหายใจไม่อิ่มขัดจังหวะการสนทนาของข้าพเจ้าอยู่เสมอ
ข้าพเจ้าไม่จำเป็นต้องพักในระหว่างวัน	0 1 2 3 4 5	ข้าพเจ้าจำเป็นต้องพักในระหว่างวันอยู่เสมอ
ข้าพเจ้าไม่รู้สึกเหนื่อย	0 1 2 3 4 5	ข้าพเจ้ารู้สึกเหนื่อยอยู่ตลอดเวลา
ข้าพเจ้ามีพละกำลังมาก	0 1 2 3 4 5	ข้าพเจ้าไม่มีพละกำลังเลย
ข้าพเจ้าเดินขึ้นบันไดหนึ่งชั้นโดยไม่มีอาการหายใจไม่อิ่ม	0 1 2 3 4 5	ข้าพเจ้าเดินขึ้นบันไดหนึ่งชั้นโดยมีอาการหายใจไม่อิ่มเป็นอย่างมาก
ข้าพเจ้ามีความมั่นใจในการออกไปในที่สาธารณะหรือที่ที่มีผู้คนพลุกพล่าน แม้ว่าข้าพเจ้ามีภาวะความดันหลอดเลือดปอดสูง	0 1 2 3 4 5	ข้าพเจ้าไม่มีความมั่นใจโดยสิ้นเชิงในการออกไปในที่สาธารณะหรือที่ที่มีผู้คนพลุกพล่าน เนื่องจากข้าพเจ้ามีภาวะความดันหลอดเลือดปอดสูง
ภาวะความดันหลอดเลือดปอดสูงไม่ส่งผลกระทบต่อการใช้ชีวิตของข้าพเจ้า	0 1 2 3 4 5	ภาวะความดันหลอดเลือดปอดสูงส่งผลกระทบต่อการใช้ชีวิตของข้าพเจ้าเป็นอย่างมาก
ข้าพเจ้าไม่ต้องพึ่งพาผู้ใด	0 1 2 3 4 5	ข้าพเจ้าต้องพึ่งพาผู้อื่นอยู่เสมอ
ข้าพเจ้าไม่เคยรู้สึกว่าตัวเองเป็นภาระ	0 1 2 3 4 5	ข้าพเจ้ารู้สึกว่าตัวเองเป็นภาระอยู่เสมอ

ชื่อ...วันที่..คะแนนรวม.................................

Translated in full with permission of the PHA by Burabha Pussadhamma and Sahachat Aueyingsak, et al., Faculty of Medicine, Khon Kaen University, Khon Kaen, Thailand. Copyright © PHA UK 2024.
แปลโดยสมบูรณ์โดยได้รับอนุญาตจาก PHA โดย บูรพา ปุสธรรม และ สหฉัตร เอื้อหยิ่งศักดิ์ และคณะ คณะแพทยศาสตร์ มหาวิทยาลัยขอนแก่น จังหวัดขอนแก่น ประเทศไทย ลิขสิทธิ์โดย PHA UK 2024. Version 1.0, กรกฎาคม 2567
